# A comparative study on mental health and adaptability between older and younger adults during the COVID-19 circuit breaker in Singapore

**DOI:** 10.1186/s12889-022-12857-y

**Published:** 2022-03-15

**Authors:** Chou Chuen Yu, Nien Xiang Tou, James Alvin Low

**Affiliations:** 1 Geriatric Education and Research Institute, Yishun Central 2, Singapore, 768024 Singapore; 2 Khoo Teck Phuat Hospital, Singapore, Singapore

**Keywords:** Aging, Coronavirus, Stress, Anxiety, Depression

## Abstract

**Background:**

While older age is associated with better emotional well-being, it is unclear whether such age advantages remain during a pandemic. This study examined differences in mental health, adaptive behaviours, social support, perceived stress, digital media usage, and perceived change in circumstances between younger and older adults during the circuit breaker period (partial lockdown) in Singapore.

**Methods:**

A door-to-door survey was administered to a nationally representative sample of 602 younger (*n* = 302) and older (*n* = 300) adults aged 21–89 years from Singapore from 17 October to 27 November 2020. All participants self-reported their depression, anxiety, stress, adaptive behaviours, social support, perceived stress, change in circumstances, and digital media usage during the partial lockdown period.

**Results:**

Older adults were found to report significantly lower levels of depression, anxiety, and stress as compared to younger adults. Although older adults were less able to perform essential activities during the lockdown, they were more adaptable psycho-socially. Logistic regression analyses revealed that for older age group, adaptability and health status significantly predicted better mental health. Older adults had higher odds of low depression scores [odds ratio (OR) 1.81, 95% confidence intervals (CI) 1.07–3.08], anxiety scores (OR 1.80, 95% CI 1.05–3.08), and stress scores (OR 3.05, 95% CI 1.72–5.41). In addition, adaptability was found to moderate the relationship between age and mental health with detrimental effects of low adaptability stronger for younger adults than older adults.

**Conclusions:**

During the lockdown period, older adults in Singapore had better mental health, perceived less stress-related concerns and were more adaptable psycho-socially as compared to younger adults. This study’s findings extend current evidence that age-related advantages in emotional well-being persisted in the wake of the COVID-19 pandemic.

## Introduction

The Singapore government implemented a partial lockdown, termed locally as ‘circuit breaker’ (CB), from 7 April to 1 June 2020 to curb growing community transmission of the COVID-19 (SARS-CoV-2) virus. The aim was to enforce social distancing measures by reducing movements and interactions in public and private places [1]. The CB measures entailed closing of all non-essential workplaces, schools, recreational venues, tourist attractions and places of worship. Essential services, such as healthcare and grocery stores, remained open to support the daily needs of the people while food establishments were only allowed to offer take-way or home delivery food. A work-from-home and telecommuting policy was also implemented at the same time. Safe distancing measures also included restrictions on the numbers of friends and family members not living together who were allowed for gatherings.

During the early stages of Singapore’s CB, there were concerns that various measures to restrict movements had taken a toll on the mental health of older adults [2]. These measures particularly affected older adults who, not only had challenges keeping up with the rapidly changing safe distancing rules but also, were less able to utilise digital platforms to meet their daily needs such as purchasing essential goods, accessing services, seeking entertainment and socialising [3]. Concerns were raised about whether older adults were less adaptable, and their mental health more adversely affected than the younger groups, during the CB period.

Previous studies indicated that older age is linked with greater emotional well-being and decreased reactivity in the face of stressors [4]. However, at the inception of this study in June 2020, there was limited data showing that age advantages in emotional well-being are maintained in the wake of the COVID-19 pandemic. Theoretical accounts on aging suggested that older adults were more adept at coping with crises through the accumulation of life experiences that build up their coping skills [5, 6]. Findings from a systematic review support such accounts and positive reappraisal, a form of coping strategy, was used more frequently by older adults compared to their younger counterparts with wide-ranging benefits on mental health [7]. Likewise, as a likely consequence of accrued social experience [8, 9], age has also been associated with better emotional regulation [10–13].

Despite the known advantages of age in emotional experience, the strength and vulnerability integration (SAVI) model posits that such age-related advantages will be attenuated when older adults are faced with inescapable negative situations [14]. According to this model, the restrictions imposed by safe distancing measures and the digital divide faced by older adults for prolonged periods of time, as in the context of the CB in Singapore, could therefore potentially attenuate any age advantages. Given the magnitude of the pandemic and concerns over potential mental health issues in older adults [15–18], it was of practical importance to examine relevant age-related attitudes and perceptions of the CB period, including the ability to conduct essential activities, use of the various digital platforms, preoccupation with stress concerns, adaptability to the stressors, availability of social support and views on health status.

Furthermore, there has been particular interest on the role of adaptability as it has been considered as a key mental resource that facilitates positive outcomes especially in a rapidly changing environment such as the present pandemic where individuals are subjected to conditions of social and physical restrictions [19]. Adaptability has been defined as “the capacity to make appropriate responses to changed or changing situations; the ability to modify or adjust one’s behaviour in meeting different circumstances” [20]. Being able to adapt successfully is important as one can plausibly recover more quickly from environmental stressors.

The primary aim of this study was to assess and compare the mental health of older and younger adults in Singapore during the CB period and determine if known age advantages would be attenuated in the context of CB. The secondary aims were to examine differences in adaptive behaviours, social support, perceived stress concerns, health status, digital media usage and change in circumstances between the two groups and the relationship between these measures and mental health. Beyond examining differences in adaptability, this study also explored if adaptability influences the postulated age effects on mental health.

## Methods

### Study design and participants

This study employed a cross sectional survey design and adult Singapore residents aged 21 years and above were recruited using the stratified sampling method. The sampling process involved stratifying by housing type (80% public and 20% private), region (5 sectors consisting of 28 postal districts that covered the whole of Singapore), age group (equal proportion of older and younger adults in each of the 5 sectors) and gender (equal proportion). Simple random sampling was employed to select 5 residents from each selected building. In line with age classification standards by World Health Organisation, older adults were defined as those 60 years and above. Those 59 years and below were defined as younger adults.

Door-to-door survey was conducted between 17 October 2020 and 27 November 2020. The questionnaire containing all the study measures including the validated DASS scale was in the English language. To be included for the study, participants must be able to speak English, which is the first language of Singapore, residing in the country during the CB period (7 April to 1 June 2020) and 21 years old and above of age. Only one resident of each household unit was approached for survey interview using computer-assisted personal interviewing (CAPI) technique. Participants were excluded if they exhibited signs of cognitive impairment. All interviewers were trained by the Geriatrician in the study team to screen for cognitive impairment. For individuals between age of 21–69 years old, they were excluded during the introduction stage and consent taking process if they exhibited signs of (i) memory loss, (ii) delirium (e.g., drowsy, sleepy, agitation) and (iii) language problems (e.g., repeating sentences that don't make sense). Additionally, individuals above 70 years old and above were excluded if they failed any one of three items from the Abbreviated Mental Test Score [21]. The purpose of the test was to rapidly screen the older adults for the possibility of dementia, mental confusion and other cognitive impairments. All interviewers involved were also trained to administer the survey questionnaire stipulated by the research team. To ensure the safety of participants and interviewers, data collection procedures complied with existing safe distancing measures during the study period. This study received ethics approval from National Healthcare Group Domain Specific Review Board (2020/00973) and all participants gave written informed consent.

### Study measures

#### Mental health

Mental health status of participants was assessed using the shortened version of Depression, Anxiety, and Stress Scale (DASS-21) [22]. DASS-21 consists of three 7-item subscales designed to measure levels of depression, anxiety, and stress. A sample item for Depression included “I couldn't seem to experience any positive feeling at all”. A sample item for Anxiety included “I was worried about situations in which I might panic and make a fool of myself”. A sample item for Stress included “I found it difficult to relax”. Internal consistencies of all three subscales were found to be good (Depression, α = 0.87; Anxiety, α = 0.76; Stress, α = 0.87). Participants indicated the extent each item statement applied to them during the CB period on a 4-point Likert scale ranging from 0 (did not apply to me at all) to 4 (applied to me very much or most of the time). Each subscale was scored by summing up the respective item scores and multiplied by two [22] and categorized into either high or low value group using the median as a cut-off. This approach of categorizing variables is justified on account of the highly skewed distributions [23, 24] with most cases obtaining the lowest possible score for each of the subscales.

#### Adaptive behaviours

Participants were asked about their adaptability in various psycho-social domains (e.g., “I was able to adjust my regular social activities to my satisfaction”, “I was able to adjust the way I interact with those I lived with to my satisfaction” and “I was able to adjust to how I spend my free time [e.g., hobbies, entertainment] to my satisfaction”) and ability to run essential activities (e.g., “I was able to physically run essential errands that I needed to do”, “I was able to use online services to settle what I needed to do [e.g. online banking, fill application forms]” and “I was able to buy takeaway food by myself if there was a need to do so”) during the lockdown period. Participants responded to a 5-point Likert scale ranging from 1 (Strongly disagree) to 5 (Strongly agree). Both scales showed good internal consistency (Adaptability, α = 0.82; Running essential activities, α = 0.81).

#### Social support

A 3-item measure adapted from the social support subscale of the Resilience Scale for Adults [25] was employed to assess the social support of participants during the lockdown period. Sample items included “I have some close friends/family members who really care about me”, “I always have someone who can help me when needed” and “I can discuss personal matter with friends/family members”. Participants responded to a 5-point Likert scale ranging from 1 (Strongly disagree) to 5 (Strongly agree). The scale was found to have good internal consistency (α = 0.86).

#### Perceived stress concerns

Participants were asked to rate their perceived stress in relation to physical health concerns, finance related concerns, emotion related concerns, supplies related concerns, news and information related concerns, as well as change of routine. Sample items in response to the following question “Thinking about the circuit breaker period please rate to what extent you agree that the following are concerns that generally affect the stress levels of yourself? included “Physical health concerns (e.g. contracting COVID-19/ deterioration of existing health/not being able to visit doctor for check-up, etc.)” and “Finance related concerns (e.g. / losing income / not paying bills/ not being able to pay rent / losing job / impact on my business, etc.)”. Participants responded to a 5-point Likert scale ranging from 1 (Strongly disagree) to 5 (Strongly agree). The 6-item scale was found to have good internal consistency (α = 0.88).

#### Digital media usage

Participants self-reported their daily average time spent on the internet during the CB period. In addition, they were asked to rate their ability in using digital platforms to get updates on COVID-19 situation, and to meet their personal needs during the CB period. Sample items included “I was able to keep up to date on all the changing measures and regulations announced by the government related to the COVID-19 situation through the various online platforms (Such as Channel News Asia, Gov.sg Telegram and Whatsapp Groups)” and “ Overall, I was able to use digital platforms to meet my needs during the circuit breaker (e.g. to buy supplies, to run essential services, entertainment such as music, video or gaming, for work, socialising etc.)”. Participants responded to a 5-point Likert scale ranging from 1 (Strongly disagree) to 5 (Strongly agree).

#### Change in circumstances

Participants were asked to rate their perceived change in circumstances in relation to their “state of health”, “financial circumstances”, “stress levels”, and “general living circumstances” during the CB period as compared to 6 months prior to the pandemic outbreak. Participants responded to a 5-point Likert scale ranging from 1 (a lot worse now) to 5 (a lot better now).

#### Other measures

Demographic data were collected on age, gender, marital status, ethnicity, religion, education levels, and occupation.

#### Sample size calculation

Based on a priori power analysis (G*Power 3.1.9.7) using a power of 0.80 and error probability of 0.05, a sample size of 300 participants is required for each group to detect a between-group difference of small effect size.

#### Statistical analysis

Independent samples *t*-tests were performed to examine differences in mental health, adaptive behaviours, social support, perceived stress, change in circumstances, and digital media usage between younger and older adults. Bivariate Pearson’s correlations were conducted to examine the relationship between age and other continuous measures. Logistic regressions were performed to ascertain the effects of age group, adaptability, social support, and self-perceived health status on the likelihood of poor mental health after adjusting for education status, gender, employment status, and digital media timespan. All analyses were conducted using Stata version 14.0 (StataCorp, Texas).

## Results

### Participant Characteristics

A total of 602 participants were recruited of which 302 are younger adults (21–59 years old, M = 39.87, SD = 11.46) and 300 were older adults (60 years old and above, M = 66.82, SD = 5.84). Majority of younger adults surveyed had completed tertiary education (70.53%) and were employed (77.49%), while older adults mostly had completed secondary level education (48.33%), had already retired (48.33%) or were not in employment (14.67%). Younger adults (34.11%) were also more likely to be single compared to older adults (7%). Demographic profiles in nationality, race, and religion were largely similar for both groups (Table 1). The sample’s breakdown by gender^a^, ethnicity^b^, nationality^c^, religion^d^, and occupation^e^ are close to population’s breakdown, indicating that our sample is representative of the population.

**Table 1:** Descriptive characteristics of participants stratified by age groups

N	All *n* (%)	Younger adults *n* (%)	Older adults *n* (%)
	602 (100.00)	302 (50.17)	300 (49.83)
Age	*M* = 53.30 *SD* = 16.26	*M* = 39.87 *SD* = 11.46	*M* = 66.82 *SD* = 5.84
Sex			
Male	302 (50.17)	133 (44.04)	169 (56.33)
Female	300 (49.83)	169 (55.96)	131 (43.67)
Marital status			
Married	431 (71.59)	188 (62.25)	243 (81.0)
Single	124 (20.60)	103 (34.11)	21 (7.00)
Divorced	22 (3.65)	11 (3.64)	11 (3.67)
Widowed	25 (4.15)	0 (.00)	25 (8.33)
Nationality			
Singaporean	565 (93.85)	274 (90.73)	291 (97.00)
Permanent Resident	37 (6.15)	28 (9.27)	9 (3.00)
Ethnicity			
Malay	91 (15.12)	49 (16.23)	42 (14.00)
Indian	82 (13.62)	43 (14.24)	39 (13.00)
Other	25 (4.16)	12 (3.97)	13 (4.33)
Religion			
Buddhism	181 (30.07)	95 (31.45)	86 (28.67)
Islam	106 (17.61)	58 (19.21)	48 (16.00)
Christianity	81 (13.46)	34 (11.26)	47 (15.67)
Hinduism	60 (9.97)	28 (9.27)	32 (10.67)
Roman Catholic	48 (7.97)	15 (4.97)	33 (11.00)
Taoism	15 (2.49)	7 (2.32)	8 (2.67)
Sikhism	3 (.50)	2 (.66)	1 (.33)
No religion	108 (17.94)	63 (20.86)	45 (15.00)
Education			
Primary and below	56 (9.30)	6 (1.99)	50 (16.67)
Secondary	200 (33.22)	55 (18.21)	145 (48.33)
Post-secondary	54 (8.97)	28 (9.27)	26 (8.67)
Tertiary and above	292 (48.51)	213 (70.53)	79 (26.33)
Occupation			
Senior Management	45 (7.48)	37 (12.25)	8 (2.67)
Professionals	93 (15.45)	79 (26.16)	14 (4.67)
Associate Professionals	62 (10.30)	43 (14.24)	19 (6.33)
Clerical Support	25 (4.15)	18 (5.96)	7 (2.33)
Sales and Services	40 (6.64)	24 (7.95)	16 (5.33)
Craftsmen	7 (1.16)	2 (.66)	5 (1.67)
Cleaners, labourers	18 (2.99)	2 (.66)	16 (5.33)
Machine Operators	20 (3.32)	7 (2.32)	13 (4.33)
Others	35 (5.81)	22 (7.27)	13 (4.33)
Not in employment	109 (18.11)	65 (21.52)	44 (14.67)
Retired	148 (24.58)	3 (.99)	145 (48.33)

### Age differences in social support, health status, digital media usage, and perceived change in circumstances

Older adults were found to report significantly poorer health and lower digital media usage as compared to their younger counterparts. However, younger adults were found to perceive relatively larger changes in circumstances compared to the past especially in the areas of finance and stress. No significant difference in social support was found between the two age groups (Table 2).

**Table 2:** Comparison of mental health, adaptive behaviours, social support, perceived stress concerns, health status, digital media usage, and change in circumstances between younger and older adults

	Younger adults		Older adults		*t*	*p*	Cohen’s *d*
	*M*	*SD*	*M*	*SD*			
**Depression**	9.40	3.19	7.75	1.73	7.89	< .01**	.64
**Anxiety**	8.29	2.16	7.49	1.21	5.61	< .01**	.46
**Stress**	9.75	3.34	8.02	1.96	7.73	< .01**	.63
**Run Essential Activity**	4.12	.56	3.81	.67	.31	< .01**	.52
**Adaptability**	3.75	.54	3.87	.48	-2.74	< .01**	.24
**Social Support**	4.31	.56	4.32	.63	-.11	.91	.00
**Perceived Stress Concerns**	3.03	.78	2.68	.80	-5.51	< .01**	.45
**Health Status**	3.47	.82	3.12	.78	5.28	< .01**	.43
**Digital media usage**							
Time spent on internet (min/day)	433.27	248.81	146.40	159.04	16.84	< .01**	1.38
Ability to use digital platforms to keep up with pandemic news	4.22	.75	3.28	1.37	10.50	< .01**	.86
Ability to use digital platforms to meet personal needs	4.17	.78	3.37	1.14	10.04	< .01**	.82
**Change in circumstances**							
Health Status	3.04	.64	3.02	.46	.43	.67	.04
Finance	2.70	.70	2.85	.58	-2.90	< .01**	.24
Stress levels	2.81	.70	2.94	.53	-2.62	< .01**	.21
General living	2.95	.57	2.96	.44	-.39	.69	.03

^**^ *p* < .01

### Relationship between age and other study variables

Table 3 presents the bivariate correlation results between age and other measures. The pattern of relationship was similar whether age is treated as categorical (older vs younger) or continuous variable. Findings showed that there was a small, negative relationship between age and ability to run essential activities. Age was also negatively associated with health status and digital media consumption, with the relationship being large for the latter.

**Table 3:** The means, standard deviation, alpha and bivariate correlations among the study variables

	*M*	*SD*	1	2	3	4	5	6	7	8	9	10	11	12
1. Age	53.30	16.26	**-**											
2. Older Adults	-	-	.83**	**-**										
			(602)											
3. Depression	8.58	2.70	-.34**	-.31**	{.87}									
			(602)	(602)										
4. Anxiety	7.89	1.80	-.21**	-.22**	.69**	{.76}								
			(602)	(602)	(602)									
5. Stress	8.89	2.87	-.25**	-.30**	.79**	.74**	{.82}							
			(602)	(602)	(602)	(602)								
6. Essential activity	4.02	.61	-.29**	-.24**	-.02	-.11	-.004	{.81}						
			(330)	(330)	(330)	(330)	(330)	(330)						
7. Adaptability	3.81	.51	.11*	.12**	-.34**	-.25**	-.34**	.41**	{.82}					
			(507)	(507)	(507)	(507)	(507)	(285)	(507)					
8. Social support	4.31	.60	-.002	.004	-.15**	-.11**	-.10*	.27**	.16**	{.86}				
			(602)	(602)	(602)	(602)	(602)	(330)	(507)					
9. Concerns	2.86	.81	-.19**	-.22**	.38**	.31**	.37**	-.10	-.20**	-.03	{.88}			
			(602)	(602)	(602)	(602)	(602)	(330)	(507)	(602)				
10. Health status	3.30	.82	-.28**	-.21**	-.09*	-.13**	-.11**	.28**	.11*	.17**	-.10*	-		
			(602)	(602)	(602)	(602)	(602)	(330)	(507)	(602)	(602)			
11. Education	4.61	2.02	-.45**	-.46**	.15**	.08*	.18**	.25**	-.07	-.02	.05	.22**	-	
			(602)	(602)	(602)	(602)	(602)	(330)	(507)	(602)	(602)	(602)		
12. Time spent on internet	290.31	253.37	-.63**	-.57**	.29**	.22**	.27**	.34**	-.07	.02	.08*	.27**	.48**	-
			(602)	(602)	(602)	(602)	(602)	(330)	(507)	(602)	(602)	(602)	(602)	

Values in {} represent internal consistency (n size)

^*^ *p* < .05, ** *p* < .01

Age was positively correlated with adaptability. This finding suggests that older adults were able to cope despite the restrictive measures. However, difference in mean scores between younger adults and older adults although significant was small in effect size [*M*_young_ = 3.75, *SD*_young_ = 0.54; *M*_old_ = 3.87, *SD*_old_ = 0.48; *t* (505) = -2.74, *p* < 0.01, *d* = 0.24]. Examining the individual items, younger adults were less likely to be able to adjust their regular social activities [*M*_young_ = 3.55, *SD*_young_ = 0.95; *M*_old_ = 3.84, *SD*_old_ = 0.71; *t* (600) = -4.31, *p* < 0.01, *d* = 0.35], fitness routines [*M*_young_ = 3.54, *SD*_young_ = 0.93; *M*_old_ = 3.76, *SD*_old_ = 0.79; *t* (600) = -3.13, *p* < 0.01, *d* = 0.25], and activities during their free time [*M*_young_ = 3.59, *SD*_young_ = 0.96; *M*_old_ = 3.82, *SD*_old_ = 0.73; *t* (600) = -3.36, *p* < 0.01, *d* = 0.05]. Effect sizes of these differences were small.

#### Main effects of age, adaptability and health status on predicting DASS scores

The logistics regression models were statistically significant for depression, *X*^2^ (8) = 91.5.0, *p* < 0.01, anxiety, *X*^2^ (8) = 71.4, *p* < 0.01 and stress, *X*^2^ (8) = 105.7, *p* < 0.01 (see Table 4 for adjusted and unadjusted models). Respectively, the models explained for 22.1%, 18% and 25.6% (Cragg & Uhler’s R^2^) of the variance in depression, anxiety and stress.

**Table 4:** Association of adaptability, social support, health status and age group with mental health

	Low Depression Scores		Low Anxiety Scores		Low Stress Scores	
	Model 1^a^ OR (95% CI)	Model 2^b^ OR (95% CI)	Model 1^a^ OR (95% CI)	Model 2^b^ OR (95% CI)	Model 1^a^ OR (95% CI)	Model 2^b^ OR (95% CI)
Adaptability	2.46 (1.63, 3.70) **	2.57 (1.69, 3.94) **	1.81 (1.23, 2.67) **	1.79 (1.20, 2.66) **	2.70 (1.78, 4.09) **	2.95 (1.92, 4.56) **
Social Support	1.19 (.87, 1.63)	1.19 (.86, 1.65)	1.13 (.82, .1.57)	1.19 (.86, .1.66)	1.16 (.84, 1.62)	1.20 (.86, 1.68)
Health status	1.38 (1.08, 1.77) *	1.57 (1.20, 2.07) **	1.74 (1.33, 2.27) **	1.91 (1.44, 2.54) **	1.61 (1.24, 2.11) **	1.78 (1.34, 2.37) **
Older Adults	3.12 (2.11, 4.60)**	1.90 (1.10, 3.31) *	3.08 (2.05, 4.62) **	1.80 (1.03, 3.12) *	3.87 (2.56, 5.85) **	3.02 (1.66, 5.46) **

High/low value was determined using the median of the logarithmic transformed scores as a cut-off: Depression -1.95; Anxiety -1.95; Stress -2.08

OR, odds ratio; 95% CI, 95% confidence interval

^*^p < .05, **p < .01

^a^Unadjusted

^b^Adjusted for education status, gender, occupation type, time on internet

Examining the variables in the models, individual adaptability, self-perceived good health and being in the older adults age group were significant predictors of lower odds of experiencing high depression, high anxiety and high stress. This was the case even after controlling for effects of gender, education, occupational type and time spent on the internet. Findings show that for older adults (vs. younger adults), the odds were lower for depression (1.90 times less likely), anxiety (1.80 times less likely) and stress (3.02 times less likely). On individual adaptability, for each unit increase in scores, the odds of depression (2.57 times less likely), anxiety (1.79 times less likely) and stress (2.95 times less likely) were lower. For each unit increase in self-perceived health score, the odds of depression (1.57 times less likely), anxiety (1.91 times less likely) and stress (1.78 times less likely) were lower. Social support was not a significant predictor in all the models.

### Interaction effects of age and adaptability on DASS

**Fig. 1:**
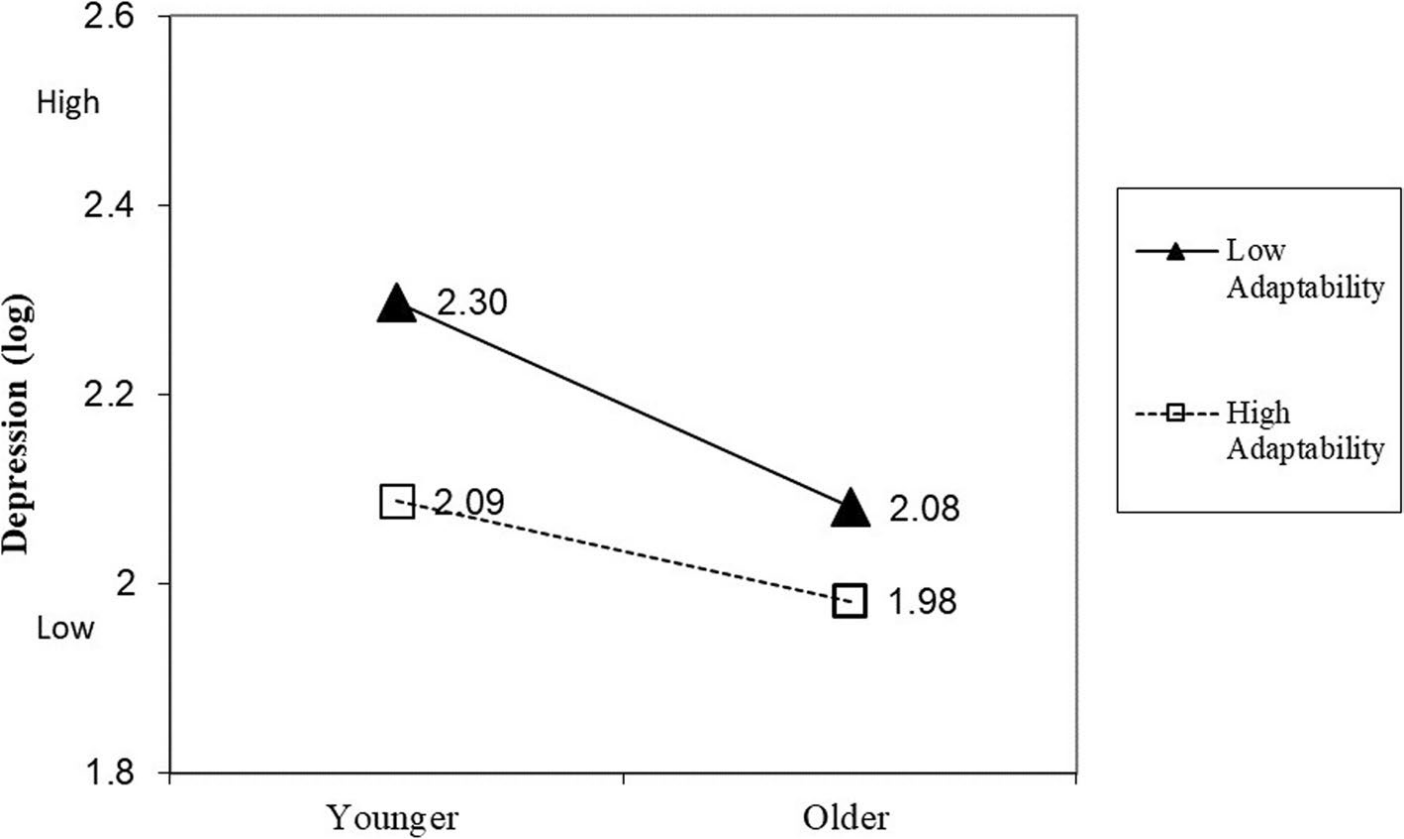
Moderator effect of adaptability on the relationship between age and depression

**Fig. 2:**
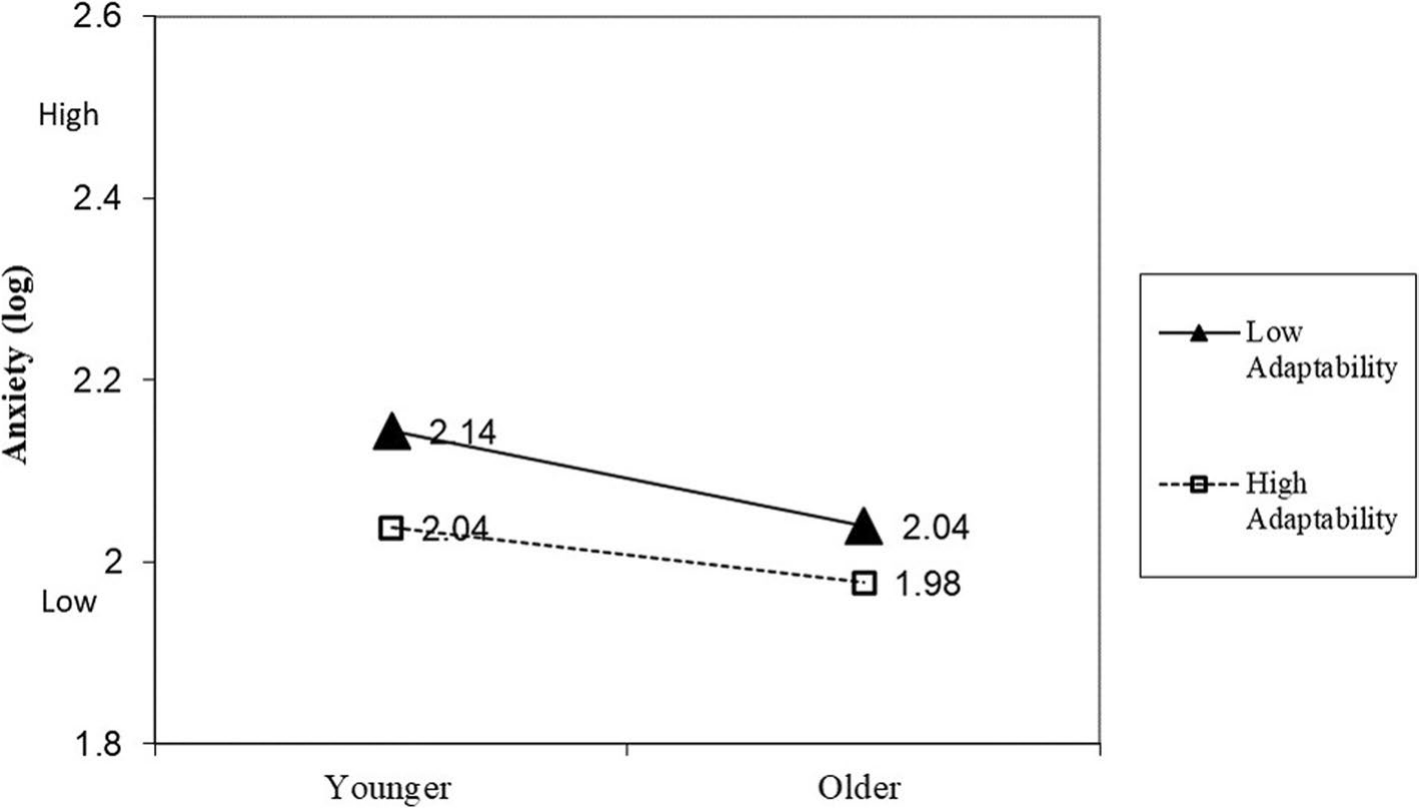
Moderator effect of adaptability on the relationship between age and anxiety

**Fig. 3:**
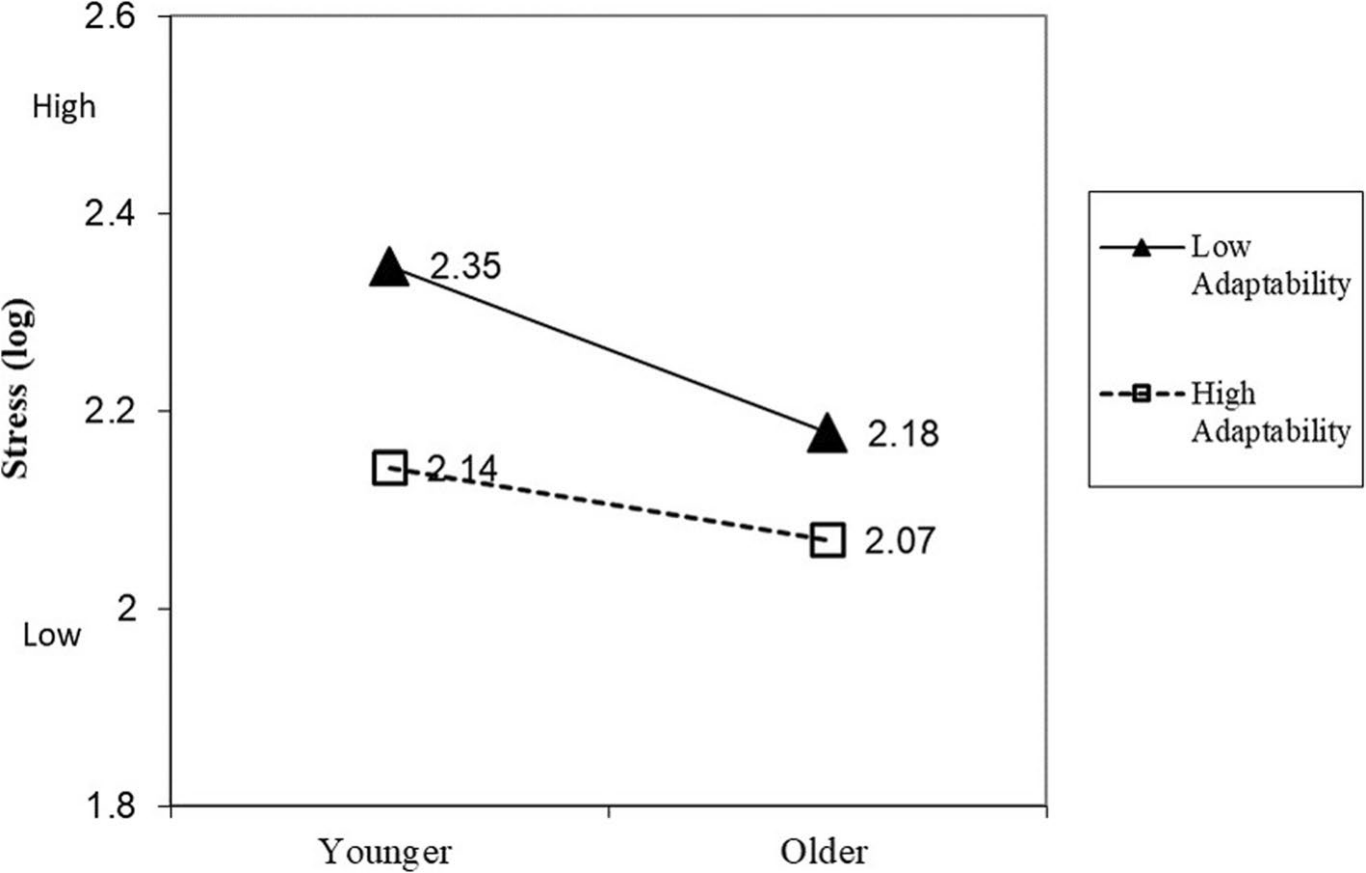
Moderator effect of adaptability on the relationship between age and stress

To explore possible moderating role of adaptability on the relationship between age (as a continuous variable) and DASS, hierarchical regression analyses were conducted. As the variables of depression, anxiety and stress did not have a normal distribution, a logarithmic transformation was performed on these variables. For the variable depression, the interaction was significant (*β* = 0.94, *p* < 0.01) and accounted for a small but significant increase in proportion of variance of 1% (*ΔR*_*2*_ = 0.01, *p* < 0.001). Simple slopes analysis (see Fig. 1) revealed that the interaction effect (non-standardized) was stronger for those low in adaptability (*b* = -0.007, *p* < 0.001) than those high in adaptability (*b* = -0.003, *p* < 0.001). For the variable anxiety, the interaction was not significant (*β* = 0.51, *p* = 0.17). Despite the non-significance, simple slopes analysis (see Fig. 2) revealed that the interaction effect (non-standardized) was in line with the expected direction and was stronger for those low in adaptability (*b* = -0.003, *p* > 0.05) than those high in adaptability (*b* = -0.002, *p* > 0.05). The statistical power attained in the study was 0.26 and the sample size (*N* = 507) of the study may have played a role in limiting the significance of the analysis. Finally, for the variable stress, the interaction was significant (*b* = 0.73, *p* = 0.04) and accounted for a small but significant increase in proportion of variance of 1% (*ΔR*_*2*_ = 0.01, *p* = . 04). Simple slopes analysis (see Fig. 3) revealed that the interaction effect (non-standardized) was stronger for those low in adaptability (*b* = -0.005, *p* < 0.001) than those high in adaptability (*b* = -0.002, *p* = 0.02).

## Discussion

This study was conceived when there were concerns that the well-being of older adults would be adversely affected during the unprecedented COVID-19 pandemic. In the context of strict movement restrictions, social isolation and safe distancing measures, the present findings suggest that age advantages in emotional experiences were maintained in community-dwelling older adults. Compared to their younger counterparts, older adults reported significantly lower levels of depression, anxiety, and stress in addition to significantly lower stress concerns. Age advantages persisted despite older adults being less able to run essential activities or leverage on digital resources during the CB period. Auxiliary analyses showed that age advantages persisted and predicted lower odds of experiencing depression, anxiety and stress even after adjusting for significant predictors such as adaptability and health status.

Regulation of emotional well-being has been purported to improve with age [26] but such advantages according to the SAVI model would diminish under inescapable negative situations [14]. This study did not find evidence that age advantages had diminished as a consequence of the restrictions imposed by the CB measures. In line with other recent studies, the association between age and mental health persisted during the pandemic [26–30].

Given the excess mortality risk of older adults [31] and tight restrictions imposed to curb the spread of COVID-19, it has been proposed that the risk of poorer mental health would be higher for older adults [32]. This risk is further compounded by the ‘digital divide’. Although this study found evidence that older adults were less able to run essential activities such as using online platforms to obtain their daily necessities or to keep up to date on the changing government’s measures and regulations related to COVID-19, it was noteworthy that older adults continued to fare better than younger adults in terms of mental health.

The auxiliary analyses conducted in this study further supports the position that age effects on mental health persist as this was found to be the case even after adjusting for other predictive variables. The findings on younger adults, which corroborated with other studies, showed that this group reported greater degree of change to their lives due to the imposed restrictive measures [33] and were less effective in coping with stressors during the pandemic [4, 34]. These observed age advantages could plausibly be explained by improved emotion regulation, which was postulated to arise from shifts in motivation and goals as an individual’s perspective of time changes [35]. Hence, older adults may potentially be less reactive to stressors as compared to younger adults [36]. Another plausible explanation could be due to compounded effects of additional stressors common to the life stage of younger adults [33]. The mean age of younger adults in this study is within the “sandwich generation” and hence it is common for these adults in Singapore to care for two generations in the family and in the context of lockdown measures in the pandemic, this would have arguably caused greater stress. As shown in this study, stressors related to finance, emotions and routines were starkly higher than older adults. Additionally, the implementation of CB measures severely disrupted several aspects of the younger adult’s lifestyle including work environment, leisure pursuits and social activities. It was also possible that younger adults had more concerns and responsibilities such as in building their careers, on top of raising a family and supporting their senior parents. In contrast, the lifestyle of older adults remained little changed and more stable, especially for many of the older adults in this study who were retired. Although a recent study suggested that spirituality and religiosity was higher in older adults and could be a protective factor during the pandemic [37], this study did not include measures that delved deeper into these domains. Group differences in religious affiliation in this study however were not found to be significant. Future study could examine in greater detail the effects of religiosity especially in a multi-cultural and multi-religious society such as Singapore.

As the pandemic has strengthened perspectives of older adults as being highly vulnerable [38], findings from this study suggest that this may not be accurate considering how older adults appeared relatively more resilient in their mental health and they continued to be able to adapt psycho-socially. Well-intended public communications aimed at protecting the health of older adults should be mindful of painting an overtly homogenous picture of older adults as being vulnerable requiring help and assistance [39]. This is important given that pandemic messaging can excessively shape the perception of vulnerability for healthy community dwelling older adults causing undue emotional responses that can impact their everyday lives [18, 40, 41]. Beyond the call for better calibration of pandemic messaging for older adults, policy makers should also focus attention on younger adults. Indeed, findings from this study, in line with others, highlighted the importance of tailoring messaging to support younger adults considering how the odds of experiencing adverse mental health (i.e., depression, stress and anxiety) was higher for them.

While the present findings were in line with recent studies [27–30], which showed that the well-being of older adults persisted during the pandemic, this study sampled only cognitively healthy community-dwelling older adults and, therefore findings may not be generalizable to other groups of such as those living in care facilities or those facing existing mental health issues such as the very frail or suffering from cognitive or sensory impairments [42–45]. There was also the possibility that age advantages were maintained because conditions imposed by partial lockdown measures may not have created the kind of high arousal state specified by the SAVI model that would impact the mental health of older adults. Nonetheless, this study included a measure showing that older adults reported stress levels worsening compared to 6 months before the pandemic (*M* = 3.06, see Table 2), hence suggesting that mental health was affected. Future research planning to model after the methodology of this study should consider including holistic measures that would be able to capture intra-individual changes in depression and anxiety.

An important finding of this study was the role of adaptability in maintaining mental well-being during the CB period. It was demonstrated that at all levels of age, those with low adaptability reported higher levels of stress and depression compared to those with high adaptability. This was the case even for older adults although low adaptability was shown to have stronger effects on stress and depression especially for younger adults. These findings suggest that policy makers can consider providing resources to help adults low in adaptability to adjust to stressful circumstances as this may build resilience. In the context of the pandemic, findings from this study suggest helping adults to adjust in some specific domains may be beneficial to their psycho-social well-being. These include (i) social activities (ii) fitness routines, (iii) spending of leisure time (iv) starting new activity to keep oneself occupied and (v) attend to activities important for one’s welfare. As this study did not examine how some adults are more adaptable than others, future research can consider investigating the reasons this is the case in the context of a pandemic since this would further inform policy making.

As the measure of adaptability was developed out of the practical need to understand how locals in Singapore adapt to the various domains, internal validity of the measure needs further testing although Cronbach Alpha was found to be good. Beyond recommending this, future research could also consider studying the effects of adaptability on mental well-being during a pandemic using other dimensions that may not have been captured in this study.

The methodological strength of the present study is the use of door-to-door surveys to reach out to community-dwelling older adults during an on-going pandemic. This approach ensures that views from adults who do not have access to digital resources are represented and add to the literature by complementing findings from other studies using different approaches (e.g., self-administered online, telephone). Moreover, interviewer administered questionnaires allowed for more accurate screening and higher quality of responses from participants. Finally, the cross-sectional survey design was appropriate considering how all adults went through the pandemic and collectively experienced the lived reality of the CB measures. The sampling approach and the use of a nationally representative sample therefore allowed inferences to be made to the wider population that other methods will find it challenging to achieve during the pandemic. This is critical for informing public policy Nevertheless, it is important to acknowledge the limitations of this study beyond the possible bias sample as already raised. Due to its cross-sectional nature, our study was not able to determine the temporality of the findings. Future longitudinal studies are warranted to examine intra-individual changes in well-being as a pandemic evolves over a longer period of time. In addition, all measures of this study were collected from participants’ retrospective recollection of past events. Although findings on age advantages on the whole mirror existing studies, this study cannot conclusively rule out the possibility that responses may be subjected to recall bias.

## Conclusion

The present study has provided insight on the mental health status of the older and younger adults in Singapore during the CB period. Our results indicated that older adults had better mental health, perceived less stress concerns and were more adaptable psycho-socially as compared to younger adults. This study helps healthcare policymakers and decision-makers better understand age-related differences of the impact of COVID-19 on mental health and wellbeing as well as inform and guide the former in formulating intervention strategies that address the multidimensional character of this pandemic, which is a crisis of monumental proportions.

## Data Availability

The datasets used and/or analysed during the current study are available from the corresponding author on reasonable request.

## Abbreviations

CB: Circuit breaker
DASS-21: Depression, anxiety, and stress scale
SAVI: Strength and vulnerability integration
